# Carbon savings associated with changing surgical trends in total knee arthroplasty in England: a retrospective observational study using administrative data

**DOI:** 10.1308/rcsann.2024.0035

**Published:** 2024-09-03

**Authors:** E Ojelade, J Koris, H Begum, M Van-Hove, TWR Briggs, WK Gray

**Affiliations:** ^1^ Getting It Right First Time Programme, NHS England, UK; ^2^ Royal National Orthopaedic Hospital NHS Trust, UK; ^3^ Oxford University Hospitals NHS Foundation Trust, UK; ^4^ Greener NHS National Programme, UK; ^5^ University of Exeter, UK

**Keywords:** Total knee replacement, Knee arthroscopy, Carbon footprint, Sustainable healthcare

## Abstract

**Background:**

Best practice pathways for common surgical procedures, including total knee arthroplasty (TKA), have the potential to improve patient outcomes and reduce carbon emissions. We aimed to estimate the reduction in carbon emissions due to changing trends in the care of patients undergoing TKA in England.

**Methods:**

This was a retrospective analysis of Hospital Episode Statistics data from 1 April 2013 to 31 March 2022 on adults undergoing elective primary TKA in England. The carbon footprint for each patient was calculated using carbon factors for multiple steps in the pathway, including ipsilateral knee arthroscopies in the year preceding the TKA, outpatient attendances, the index TKA, revisions of the TKA performed within 180 days of the index procedure, length of hospital stay and emergency readmissions.

**Results:**

A total of 648,861 TKA operations were identified. Over the study period, the median length of stay reduced from four to three days, the proportion of patients undergoing ipsilateral knee arthroscopies performed within a year before TKA surgery fell from 5.9% to 0.5% and the number of early revisions and emergency readmissions also fell. The per-patient carbon footprint reduced from 378.8kgCO_2_e to 295.2kgCO_2_e over this time. If all the study patients had the same carbon footprint as the average patient in 2021/2022, 32.4kilotons CO_2_e would have been saved, enough to power 29,509 UK homes for one year.

**Conclusions:**

Practices that were introduced primarily to improve patient outcomes can contribute to a reduction in the carbon footprint.

## Introduction

Climate change is thought to represent the largest health threat of the 21st century.^1^ With extreme weather events, such as heatwaves and flooding, and increasing risk of air pollution, it is estimated that 1 in 20 deaths in the UK may have climate change as a contributory factor.^2^ Global healthcare is a significant contributor to climate change, both in carbon emissions and waste. It is estimated that 5% of all global emissions are directly or indirectly from healthcare provision.^3^ Only four countries in the world produce more emissions that pollute the environment than that produced by global healthcare.^3^

The UK NHS is responsible for 4.6% of all UK carbon emissions.^4,5^ Tackling this footprint is essential to achieve government net zero goals but will also improve the health of the nation. Steps have been taken to reduce NHS carbon production: the ‘Delivering a net zero NHS’ report, published in summer 2022, committed to delivering net zero healthcare by 2045.^4^ This target requires the involvement and cooperation of many teams, including clinicians, management, estates, facilities, travel and transport, commissioning and procurement.^5^

Impacts on carbon footprint should be assessed as part of any changes in practice or service organisation, including initiatives aimed at improving patient care. In England, improvements to clinical care at a system level falls under the remit of the Getting It Right First Time (GIRFT) programme. GIRFT is a clinically led and data-driven initiative in NHS England that aims to reduce unwarranted variation in healthcare provision. GIRFT started its programme in orthopaedics and there have been a number of key reductions in variations made across the specialty as evidenced in the latest GIRFT report and in other studies.^6–9^ Positive changes include reductions in length of stay, reduction in knee arthroscopy in the year preceding a total knee arthroplasty (TKA), and reduced rates of revision one year postoperatively. Although these changes cannot be attributed solely to the GIRFT programme, it is likely that GIRFT contributed to changing the mindset and practices of clinicians in England.

The aim of this study was to estimate the potential amount of carbon release saved by changing trends in the care of patients undergoing TKA in England. A secondary aim was to estimate the potential future carbon savings if we make these changes across all providers in England.

## Methods

### Study design

This was a retrospective analysis of administrative data from the Hospital Episode Statistics (HES) database for England. The HES database is maintained by NHS England. All NHS hospital activity, including activity at non-NHS sites where the patient is funded by the NHS, is entered by trained clinical coders in each trust and submitted to NHS England for cleaning and collation.

### Ethics

The presentation of data follows NHS England guidance for use of HES data for research purposes.^10^ Individualised consent was not required for analysis, and no patient-identifiable data were used. Reporting follows current guidelines for observational studies.^11^

### Data collection

The data presented are for adult (≥17 years) patients undergoing primary, elective TKA for the nine financial years 2013/2014 to 2021/2022 (inclusive). We identified primary, elective TKA procedures if any of the following Office of Populations Censuses and Surveys Classification of Interventions and Procedures version 4 (OPCS-4) codes were present in the primary position of the procedural record: O181 or O188 or O189 or W401 or W408 or W409 or W411 or W418 or W419 or W421 or W428 or W429. The definitions of these OPCS-4 codes are given in Supplementary material Table S1.

### Inclusion and exclusion criteria for index procedure

Patients were excluded where:

1. The procedure was nonelective.
2. The patient was aged <17 years.
3. Any of the OPCS-4 or International Statistical Classification of Diseases and Related Health Problems, tenth edition (ICD-10) codes given in Supplementary material Table S1 were present in the patient record for their index episode of care.

The data extraction process is summarised in Supplementary material Figure S1.

### Defining the carbon footprint for TKA procedures

Detailed data on the carbon footprint of the GIRFT TKA surgical pathway in England are not currently available.^12^ However, we used the best estimates that were available for England from the Greener NHS and Sustainable Healthcare Coalition's (SHC) carbon footprint data.^12^ We also only considered key elements of the idealised TKA surgical pathway as defined by the GIRFT High Volume Low Complexity programme.^13^ How each of these elements was defined and the carbon factors applied to each element is described below:

1. A noncomplex surgical procedure was estimated to release 35.1kgCO_2_e.^14^ This figure was used for primary (index) TKA, ipsilateral revision TKA within 180 days of the index procedure and ipsilateral arthroscopies in the year before the index procedure. Primary TKA procedures were identified as described above. Revision TKA procedures were defined using any of the OPCS-4 codes listed in Supplementary material Table S2. Arthroscopy procedures were defined using any of the OPCS-4 codes listed in Supplementary material Table S3.
2. A low-intensity inpatient bed day was estimated to release 37.9kgCO_2_e.^15^ This figure was used for days of hospital ward stay for primary TKA, ipsilateral revision TKA and ipsilateral arthroscopies.
3. A high-intensity inpatient bed day was estimated to release 89.5kgCO_2_e.^15^ This figure was used for days of critical care stay for primary TKA and ipsilateral revision TKA.
4. Emergency department attendance was estimated to release 13.8kgCO_2_e.^16^ This figure was used for any emergency readmissions within 30 days of discharge from the index procedure and added to the figure for the number of days in a low and high-intensity bed for the emergency readmission, as described in (2) and (3) above.
5. Outpatient appointments in the year before and the year after index TKA surgery. A face-to-face outpatient appointment was estimated to release 22.0kgCO_2_e, whereas a virtual appointment was estimated to release 0.1kgCO_2_e.
6. Return travel was estimated to release 4.4kgCO_2_e. This figure was applied to primary and revision operations and emergency readmissions. Travel is included in the overall figure for outpatient attendances.

### Other data extracted

The following data were also extracted.

1. Age in years at time of operation. Age was categorised as <50 years, 50–59 years, 60–69 years, 70–79 years and ≥80 years based on visual inspection of the data to ensure categories were of broadly similar sizes.
2. Sex.
3. Financial year of admission.
4. Hospital Frailty Risk Score (HFRS) categorised into mild, moderate, severe, and none based on established categories.^17^
5. Index of Multiple Deprivation (IMD) quintile based on national percentiles for Lower Super Output Areas (LSOAs) in England.^18^
6. Mortality within 180 days of the primary procedure. This is reported but was not considered a relevant outcome within the scope of this paper. Mortality data were taken from the UK Office for National Statistics and linked at a patient level.

### Data management and statistical analysis

Data were analysed using a secure server controlled by NHS England. Standard statistical software including Microsoft Excel (Microsoft Corp, Redmond, WA, USA), Stata (Stata Corp LLC, College Station, TX, USA) and Alteryx (Alteryx Inc, Irvine, CA, USA) were used in the analysis of the data.

Data were summarised using standard descriptive statistics (e.g. mean, median, frequency). On visual inspection of the data, age was non-normally distributed and therefore presented as median and interquartile range.

Carbon factors were calculated at patient level and aggregated as appropriate. An estimated total carbon reduction was estimated using the per-patient carbon footprint for the 2021/2022 financial year as a reference. To provide context, CO_2_e emissions were converted to the amount of CO_2_e required to power an average UK home with electricity for one year using a conversion factor of 1,098.9kgCO_2_e per home per year.^19,20^

## Results

The data extraction process is summarised in Supplementary material Figure S1 and identified 648,861 patients undergoing TKA operations during the study period. The profile of the patients and their outcomes are summarised in Table 1. Outcomes are presented for each of the nine years of the study in Table 2. Patient numbers were relatively stable before the start of the COVID-19 pandemic in early 2020, at which point all elective surgery in England was suspended for a period of time.^21^ Patient numbers recovered somewhat during 2021/2022. In general, outcomes improved over the study period, although adverse outcomes were relatively uncommon. Rates of early revision fell from 0.4% to 0.2% across the nine years, although low rates in the post-COVID-19 period may partly reflect lower surgery volumes and longer waiting lists for revision surgery. Median length of stay declined by a day. The median number of pre- and postsurgery outpatient attendances was relatively constant over time. The number of knee arthroscopies performed in the year before primary TKA fell substantially, from 5.9% in 2013/14 to 0.5% in 2021/22. The decline in arthroscopy rates was consistent across all years, with no clear association with the COVID-19 pandemic suspension of elective surgery.^21^

**Table 1:** Characteristics of, and outcomes for, patients who had a total knee replacement between April 2013 and March 2022

Variable	Number of patients (*n* = 648,861)
Age band	
<50 years	12,510 (1.9%)
50–59 years	82,781 (12.8%)
60–69 years	212,553 (32.8%)
70–79 years	249,561 (38.5%)
≥80 years	91,456 (14.1%)
Sex (missing = 120)	
Male	372,065 (57.3%)
Female	276,676 (42.6%)
HFRS band	
None	402,642 (62.1%)
Mild	201,103 (31.0%)
Moderate	42,557 (6.6%)
Severe	2,559 (0.4%)
IMD quintile (missing = 6,776)	
1 (most deprived)	95,657 (14.9%)
2	117,622 (18.3%)
3	140,306 (21.9%)
4	147,572 (23.0%)
5 (least deprived)	140,928 (22.0%)
Median days of index stay (IQR)	3 (3–4)
Critical care admissions during index stay	9,326 (1.4%)
Emergency readmission within 30 days*	22,319 (3.4%)
Deaths within 180 days	2,673 (0.4%)
Revisions within 180 days	2,225 (0.3%)
Arthroscopies in the year before index TKA	19,757 (3.0%)
Median number of face-to-face orthopaedic outpatient attendances in the year before surgery (IQR)	3 (2–4)
Median number of face-to-face orthopaedic outpatient attendances in the year postsurgery (IQR)	2 (1–4)

*Emergency readmissions were recorded only where there was an overnight stay. HFRS = hospital frailty risk score; IMD = index of multiple deprivation; IQR = interquartile range; TKA = total knee arthroplasty.

**Table 2:** Change in proportion of patients undergoing TKA and outcomes over the nine-year study period

Financial year	2013/2014	2014/2015	2015/2016	2016/2017	2017/2018	2018/2019	2019/2020	2020/2021	2021/2022
Number of patients	74,705	79,136	80,693	84,767	79,701	81,504	78,987	26,018	63,350
Median days of index stay (IQR)	4 (3–5)	4 (3–5)	4 (3–5)	3 (3–5)	3 (3–4)	3 (2–4)	3 (2–4)	3 (2–3)	3 (2–3)
Critical care admissions during index stay	1,158 (1.6%)	1,199 (1.5%)	1,366 (1.7%)	1,361 (1.6%)	931 (1.2%)	1,241 (1.5%)	1,015 (1.3%)	273 (1.0%)	782 (1.2%)
Emergency readmission within 30 days*	2,823 (3.8%)	2,924 (3.7%)	2,799 (3.5%)	2,885 (3.4%)	2,616 (3.3%)	2,805 (3.4%)	2,717 (3.4%)	770 (3.0%)	1,980 (3.1%)
Deaths within 180 days	318 (0.4%)	326 (0.4%)	336 (0.4%)	332 (0.4%)	305 (0.4%)	338 (0.4%)	346 (0.4%)	127 (0.5%)	245 (0.4%)
Revisions within 180 days	328 (0.4%)	311 (0.4%)	302 (0.4%)	322 (0.4%)	257 (0.3%)	271 (0.3%)	187 (0.2%)	59 (0.2%)	188 (0.3%)
Arthroscopies in year before index surgery	4,424 (5.9%)	4,039 (5.1%)	3,522 (4.4%)	2,848 (3.4%)	2,065 (2.6%)	1,412 (1.7%)	985 (1.2%)	169 (0.6%)	293 (0.5%)
Median number of face-to-face orthopaedic outpatient attendances in year before surgery (IQR)	3 (2–4)	3 (2–4)	3 (2–4)	3 (2–4)	3 (2–4)	3 (2–4)	3 (2–5)	3 (1–4)	3 (1–4)
Median number of face-to-face orthopaedic outpatient attendances in year postsurgery (IQR)	3 (2–4)	2 (2–4)	2 (1–4)	2 (1–4)	2 (1–4)	2 (1–4)	2 (1–3)	2 (1–4)	2 (1–4)

*Emergency readmissions were recorded only where there was an overnight stay. IQR = interquartile range; TKA = total knee arthroplasty.

The changes in the estimated carbon footprint for the key elements of the TKA pathway over the nine-year study period are presented in Table 3. The per-patient carbon footprint associated with the index procedure fell by around 30% from 2013/2014 to 2021/2022, with the fall in length of stay the major contributor to this decline. The relative fall in the per-patient carbon footprint associated with revisions was slightly larger at almost 50% over the study period, but small in absolute terms. Likewise, the fall in the per-patient carbon footprint associated with presurgery arthroscopy was small in absolute terms, but large in relative terms, falling from 2.7kgCO_2_e to 0.2kgCO_2_e over the nine years. Declines in the per-patient carbon footprint associated with emergency readmissions and outpatient attendances were more modest, with outpatient attendances falling most noticeably from 2018/2019 onwards. The cumulative carbon saving across all years for all five carbon factors combined had all patients had a carbon footprint the same as the 2021/2022 average would have been 32,427,965.8 (32.4kilotons) kgCO_2_e, equivalent to powering 29,509 UK homes for one year.

**Table 3:** Change in proportion of patients undergoing TKA and outcomes over the nine-year study period

	2013/2014	2014/2015	2015/2016	2016/2017	2017/2018	2018/2019	2019/2020	2020/2021	2021/2022
Number of patients	74,705	79,136	80,693	84,767	79,701	81,504	78,987	26,018	63,350
Per-patient carbon footprint of index surgery (kgCO_2_e)	222.3	214.6	208.5	201.2	193.1	186.8	176.7	155.5	158.3
Per-patient carbon footprint of emergency readmissions within 30 days (kgCO_2_e)*	10.5	10.3	10.3	9.7	9.1	9.6	9.3	7.9	8.9
Per-patient carbon footprint of revisions within 180 days (kgCO_2_e)	1.4	1.3	1.1	1.1	0.9	0.9	0.8	0.3	0.8
Per-patient carbon footprint of arthroscopy in the year before index surgery (kgCO_2_e)	2.7	2.3	2.0	1.5	1.1	0.8	0.6	0.3	0.2
Per-patient carbon footprint of orthopaedic outpatient attendances in year before surgery (kgCO_2_e)	72.4	73.0	74.1	73.3	74.6	75.4	77.1	69.5	67.2
Per-patient carbon footprint of orthopaedic outpatient attendances in year postsurgery (kgCO_2_e)	69.5	68.3	67.5	66.9	67.2	67.9	56.9	58.7	59.9
Total carbon footprint (kgCO_2_e)	378.8	369.8	363.5	353.8	346.0	341.4	321.3	292.3	295.2

*Emergency readmissions are recorded only where there was an overnight stay. TKA = total knee arthroplasty.

**Figure 1:**
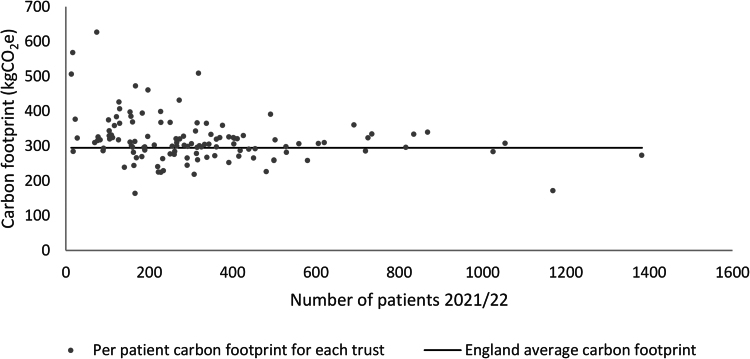
Average per-patient carbon footprint relative to patient numbers for each NHS hospital trust in 2021/2022.

Figure 1 shows the average per-patient carbon factor for each NHS hospital trust in England in 2021/2022; trusts operating on fewer than ten patients were excluded. Of the 121 trusts, the highest and lowest average total per-patient carbon footprint were 627.3kgCO_2_e and 164.1kgCO_2_e, respectively. There was evidence of a moderate and significant inverse relationship between the average per-patient carbon footprint and number of patients operated on at each trust (Spearman’s *r*=−0.262, *p*=0.004). Of these 121 trusts, if all trusts not achieving the lower quartile carbon footprint of 284.0kgCO_2_e in 2021/22 had achieved this benchmark then an estimated 1,204,730.3kgCO_2_e (1.2kilotons) could have been saved in 2021/2022, equivalent to powering 1,096 UK homes for one year.

## Discussion

We present data on the estimated savings in carbon emissions already achieved through changes in practice for TKA surgery. We also highlight the potential carbon reduction benefits of further improvements. As well as helping to reduce the carbon footprint of the NHS, these changes are associated with per-patient reductions in the financial cost of surgery and more efficient working practice. Our data provide evidence that reduced length of stay and reductions in the use of pre-TKA surgery arthroscopy have not been associated with any increase in adverse events. Similar findings have been reported in urological surgery.^22^ Rates of revision TKA and early emergency readmission declined over the study period. The reduction in rates of knee arthroscopy in the year preceding TKA was recommended by the GIRFT national report for orthopaedic surgery. The changes are welcomed given the evidence that, in patients with knee arthritis and indications for TKA, the procedure is of little benefit.^8^

Minimising hospital stay is one way to reduce carbon emissions, and there is evidence that reduced length of stay across many different specialties and procedures has no negative impact on clinical outcomes.^6,23–26^ However, data on outcomes should be monitored regularly as average length of stay continues to fall. Clinical improvement initiatives, such as NHS England’s Model Health System and National Consultant Information Programme platforms, which feedback outcomes data to trusts and surgeons, respectively, will support this monitoring.^27,28^ Adding carbon footprint data to both platforms would allow trusts and surgeons to monitor the environmental impact of their practice and pathways.

One potential source of further reduction to the carbon footprint of TKA is to reduce the number of outpatient appointments that patients require both before and after their operation. This could be achieved by considering the requirement for face-to-face appointments and replacing these with virtual appointments where appropriate, minimising unnecessary preoperative appointments by coordination of all preoperative assessment into a single visit and increasing use of patient-initiated follow-up to surgery.

We are conscious that while demand for elective surgery in England continues to exceed capacity, these trends will not achieve net zero. Beds and outpatient slots that become free will be filled by other patients. However, once an equilibrium is reached, the per-patient carbon footprint saving will be realised. Although we have considered the macro-trends in TKA, there are many other trends that could be investigated with a view to reduce carbon emissions. More detailed analysis of the carbon footprint of individual aspects of operating theatre practice is needed. It has been estimated that 42.3% of the carbon emissions associated with surgical care were due to the choice of anaesthetic gases.^29^ Further work is required focusing on choice of anaesthetic and whether increased utilisation of regional anaesthesia could help drive carbon emission reduction.

The GIRFT High Volume Low Complexity programme is a key part of the recovery of elective surgical provision following the pandemic. By reducing variation in the surgical pathways across the NHS in England, efficiency savings can be made, supporting recovery of elective services.^13^ Our study suggests that such pathways can lead to good patient outcomes while being both resource and carbon efficient.

### Strengths and limitations

This study includes a large dataset covering all TKA operations that occurred over nine years, allowing us to identify macro trends. We expect that the dataset is representative of all patients undergoing TKA in England. The HES dataset includes the ability to identify the same patient presenting at different hospitals. This is valuable in allowing more complete identification of preoperative procedures, postoperative readmissions and outpatient attendances in cases where patients may have travelled beyond their local hospital for surgery.

There are some limitations to our study. We were able to investigate the carbon footprint only for elements of the surgical pathway where data and a carbon factor were available. Referral procedures and the carbon footprint of individual elements of index TKA, arthroscopies and revision TKA were not considered and will vary considerably from one patient to another depending on complexity of presentation, patient demographics and comorbidity and individual subtleties of clinical practice of each surgical team and service organisation at each trust. Developing carbon factors that can be adjusted to suit the characteristics of each procedure would be an important step forward. Issues around data quality and consistency in HES have been highlighted.^30,31^ However, for common, planned procedures, such as elective TKA, such errors are likely to be relatively rare.

## Conclusions

This study has estimated a substantial reduction in per-patient carbon emissions with changing practice for TKA in England and identifies areas for future improvements. Carbon-efficient surgical pathways can support the NHS in England in meeting its goal of being net zero by 2045. Climate change is a major environmental risk and, as we look to improve services, we should consider the environmental impact as well as the impact on clinical care, service efficiency and patient outcomes. In many cases these outcomes will be well aligned.
